# Carotid-Threatening Penetrating Oropharyngeal Trauma in a Pediatric Patient: A Case Report and Literature Review

**DOI:** 10.7759/cureus.70170

**Published:** 2024-09-25

**Authors:** Alyssa J Snyder, Sarah McKay, Luke Mammen, Lara K Reichert

**Affiliations:** 1 Otolaryngology - Head and Neck Surgery, Albany Medical College, Albany, USA; 2 Otolaryngology - Head and Neck Surgery, Albany Medical Center, Albany, USA

**Keywords:** penetrating injury, otolaryngology case report, oropharyngeal trauma, internal carotid artery (ica), head and neck trauma, foreign body radiology

## Abstract

Penetrating oropharyngeal injury is a relatively common occurrence in pediatric patients; however, cases involving close proximity to critical vascular structures, such as the internal carotid artery (ICA), are exceedingly rare and pose significant risks. This case report describes a seven-year-old male who sustained penetrating oropharyngeal trauma with startling proximity to the ICA after being pushed into a locker with a pencil in his mouth. Initial evaluation showed the wooden pencil protruding from the soft palate without active bleeding. CT angiography revealed that it was less than 1 mm from the left ICA, with no signs of extravasation or dissection. The pencil was removed atraumatically under ketamine sedation, and the puncture site was irrigated without complications. In patients presenting with penetrating oropharyngeal trauma, injury to the internal carotid or adjacent vessels with possible resulting extravasation or dissection should be considered. This is a single case report accompanied by a literature review.

## Introduction

Penetrating oropharyngeal trauma is commonly seen in pediatric otolaryngology practices. These impalement injuries account for 1%-2% of all pediatric trauma and typically arise in pediatric males with a mean age of four years old [1,2]. The majority of penetrating oropharyngeal injuries occur when accidentally falling onto an object held in the mouth, with frequent soft palate involvement and less common hard palate involvement [3]. The most common penetrating objects in children include toothbrushes, cylindrical toys, and chopsticks [3]. Children are at an increased risk for penetrating oropharyngeal injuries due to their inclination to put foreign objects in their mouths, with the increased probability of falling and accidental injury.

The internal carotid artery (ICA) is in a close anatomical position to the posterior oropharynx, causing it to be at risk for penetrating injury. The ICA of younger patients is located more medially within the neck, resulting in closer proximity to the oropharynx than in adults [4]. This position increases the anatomical susceptibility for ICA injury in the pediatric population, putting the child at risk for vascular injury including dissection, thrombosis, and possible stroke. However, even with this increased risk, the incidence of ICA trauma due to oropharyngeal penetrance is infrequent.

Although most cases of oropharyngeal trauma can be managed conservatively with no complications, neurological and vascular complications have been reported to arise between three and 60 hours post-injury [1,5]. Some of these complications, depending on the clinical scenario, can significantly increase the risk of mortality [6]. Here, we present a rare incidence of intraoral pencil penetration threatening the internal carotid artery.

## Case presentation

**Figure 1:**
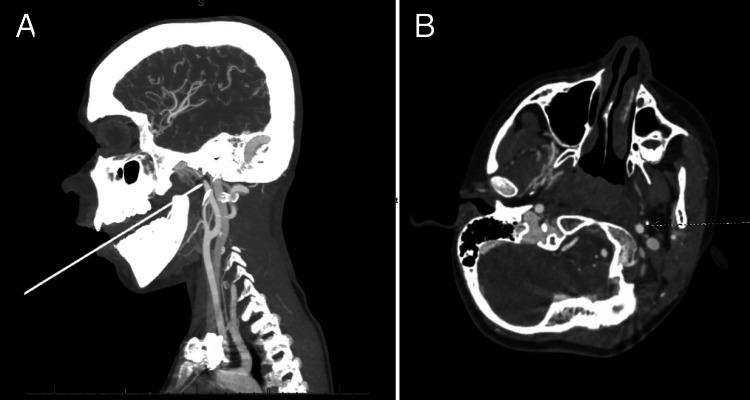
CT angiography demonstrating penetrating oropharyngeal trauma with close proximity to the internal carotid artery (A) Sagittal view of the head and neck showing the penetrating trajectory of the pencil entering the oral cavity and penetrating through the left oropharynx tissue, passing through the medial pterygoid, and continuing into the posterior aspect of the parapharyngeal fat space. (B) Axial view demonstrating the pencil (indicated by the dotted arrow) ending 0.9 mm anterolateral to the internal carotid artery without evidence of vascular extravasation or dissection. CT: computed tomography

A previously healthy seven-year-old male without significant past medical history presented to the emergency department with a traumatic penetrating oropharyngeal injury. He had a pencil in his mouth at school when he was pushed from behind and fell into his locker, resulting in the pencil becoming lodged in his left soft palate. Upon presentation to the emergency department, he was evaluated immediately by the trauma, pediatric surgery, and otolaryngology services. On the initial examination, he was hemodynamically stable, and a wooden pencil was noted protruding from the mouth, penetrating his left soft palate without any active bleeding noted. He was immediately taken for a CT angiography of the head and neck, which revealed a pencil entering the oral cavity and penetrating the left oropharynx tissue and terminating 0.9 mm anterolateral to the ICA (Figure 1). There was no evidence of active extravasation or dissection.

**Figure 2:**
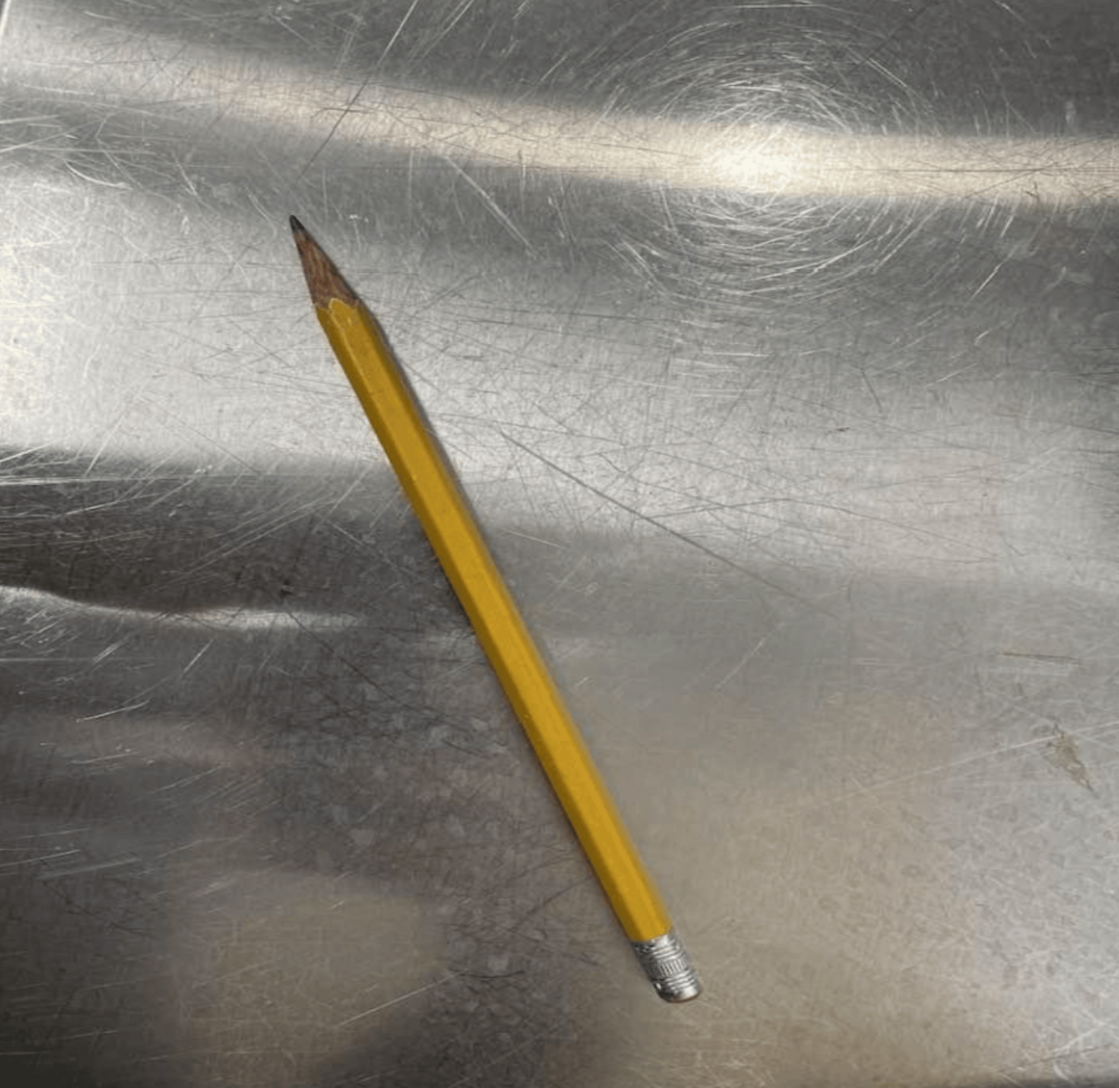
Wooden pencil post-surgical removal The wooden pencil was removed from the patient intact.

Given the proximity to the ICA and concern for possible underlying injury, the patient was urgently taken to the operating room with otolaryngology and pediatric anesthesia. Under ketamine sedation, the patient's oral cavity was examined with the pencil entrance wound posterior to the third left maxillary molar. The pencil was grasped and removed intact with minimal bleeding (Figure 2). The puncture site was copiously irrigated. Anesthesia was then reversed, and the patient was taken to the pediatric post-anesthesia care unit in stable condition.

On postoperative day 0, the patient was in stable condition and tolerating oral intake. He was discharged on chlorhexidine gluconate rinse twice daily and ibuprofen as needed for pain management. He was able to return to school and continue his diet as tolerated. He had no vascular or neurological sequelae.

## Discussion

Despite penetrating oropharyngeal trauma being common in the pediatric population, the majority of cases are minor, with rare occurrences of intraoral penetration extending into close proximity to the ICA. This case describes a rare occurrence of intraoral penetration of the soft palate with foreign body extension threatening internal carotid artery injury by less than 1 mm, requiring deliberate management. Previous literature describes the incidence and treatment of oropharyngeal soft palate trauma, with differing recommendations regarding imaging and observation time.

The majority of pediatric oropharyngeal trauma is benign and treated conservatively without complication. Out of 107 patients with traumatic oropharyngeal injuries presenting to Children's Hospital of Pittsburgh between 1998 and 2004, most oropharyngeal injuries did not require surgical intervention, with only four patients requiring foreign body removal [7]. The same study found the incidence of ICA injury in children with oropharyngeal trauma to be approximately 0.6%, including penetrating and blunt injuries [7]. The rarity of this event spurs debate regarding the management and follow-up of this injury.

Despite literature only having reported 32 cases of soft palate penetration resulting in further complication, these cases have had devastating consequences [8]. Sixteen cases of soft palate lacerations caused an intimal tear of the internal carotid artery, resulting in neurological sequelae with five deaths [9]. These cases are often complicated due to diagnostic and therapeutic challenges. One case reports a left internal carotid dissection treated conservatively with aspirin initiation [6]. Although the patient had an early and accurate diagnosis, resultant stroke occurred four days later, showing that although rare, complication is still possible days after initial injury [6].

An important component to consider when managing patients who present with similar injuries is the increased craniofacial ratio in the pediatric population [4]. Profuse bleeding from deep soft tissue and close anatomical proximity of major vessels has been reported during removal of intraoral penetrating foreign bodies [10]. Deliberate management and awareness of an operation's inherent increased difficulty due to anatomical differences is crucial.

Due to the potential of life-threatening events, previous literature suggests pediatric patients with oropharyngeal injury be observed for a period of 48-72 hours [11]. The practicality and effectiveness of this standard has been evaluated through case series and reports. On a review of 131 cases of pediatric impalement injuries of the soft palate and oropharynx, no complications were found during hospitalization or follow-up management, indicating that these patients can be managed on an outpatient basis to avoid unnecessary hospital admissions [1]. Instead, parental counseling and close follow-up could be more reasonable without sacrificing safety [1].

However, the heterogeneity of the mechanisms of injury, as well as variability in clinical presentation, does not allow for the development of robust guidelines for injuries requiring surgical intervention. There is a significant knowledge gap in evidence-based diagnosis and management of internal carotid artery injury from palatal penetration. Regardless of the severity or size of penetration, many recommend imaging to rule out vascular injury, despite varying opinions regarding the amount of observation time [7,12,13]. Fortunately, our case avoided vascular extravasation or dissection through careful removal of the foreign body after visualization using CT angiography. Further study is needed to develop standardized observation and intervention guidelines for pediatric patients presenting with similar complex cases.

## Conclusions

In conclusion, this case highlights a rare incidence of pediatric oropharyngeal penetrating trauma with dangerously close proximity to the internal carotid artery. Herein, we demonstrate the careful surgical intervention that was required to ensure no further development of vascular injury during surgery. Additionally, this report discusses the critical considerations required in managing complex pediatric presentations. Preoperative planning and meticulous intraoperative intervention are necessary to navigate these high-risk surgical cases.
